# Early IFN-Gamma Production after YF 17D Vaccine Virus Immunization in Mice and Its Association with Adaptive Immune Responses

**DOI:** 10.1371/journal.pone.0081953

**Published:** 2013-12-06

**Authors:** Patrícia C. C. Neves, Juliana R. Santos, Luciana N. Tubarão, Myrna C. Bonaldo, Ricardo Galler

**Affiliations:** 1 Vice-diretoria de Desenvolvimento Tecnológico, Instituto de Tecnologia em Imunobiológicos, Fundação Oswaldo Cruz, Rio de Janeiro, Brazil; 2 Laboratório de Biologia Molecular de Flavivírus, Instituto Oswaldo Cruz, Fundação Oswaldo Cruz, Rio de Janeiro, Brazil; Instituto Butantan, Brazil

## Abstract

Yellow Fever vaccine is one of the most efficacious human vaccines ever made. The vaccine (YF 17D) virus induces polyvalent immune responses, with a mixed T_H_1/T_H_2 CD4^+^ cell profile, which results in robust T CD8^+^ responses and high titers of neutralizing antibody. In recent years, it has been suggested that early events after yellow fever vaccination are crucial to the development of adequate acquired immunity. We have previously shown that primary immunization of humans and monkeys with YF 17D virus vaccine resulted in the early synthesis of IFN-γ. Herein we have demonstrated, for the first time that early IFN-γ production after yellow fever vaccination is a feature also of murine infection and is much more pronounced in the C57BL/6 strain compared to the BALB/c strain. Likewise, in C57BL/6 strain, we have observed the highest CD8^+^ T cells responses as well as higher titers of neutralizing antibodies and total anti-YF IgG. Regardless of this intense IFN-γ response in mice, it was not possible to see higher titers of IgG2a in relation to IgG1 in both mice lineages. However, IgG2a titers were positively correlated to neutralizing antibodies levels, pointing to an important role of IFN-γ in eliciting high quality responses against YF 17D, therefore influencing the immunogenicity of this vaccine.

## Introduction

Yellow Fever vaccine was developed during 30´s by Max Theiler and cols and has been in current use for human vaccination since it. Generally, it confers long term protection after only a single dose and more than 540 million doses have been administered globally [1]. Vaccination with YF 17D virus induces polyvalent immune responses, with a mixed T_H_1/T_H_2 CD4^+^ cell profile, which results in robust T CD8^+^ responses and high titers of neutralizing antibody [2]. Despite these characteristics, until few years ago, very little was known about the cellular and molecular mechanisms that lead to such good immune response. It is now becoming clear that early events following YF 17D immunization have a key role in determining the strength and quality of the adaptive immune response [3–7]. 

We have previously shown that primary immunization of humans with YF 17DD virus vaccine resulted in the early synthesis of IFN-γ [7], possibly mediated by NK cells. Furthermore, PBMC from rhesus monkeys vaccinated with YF 17D virus were also shown to produce IFN-γ early after vaccination, before the appearance of adaptive immune responses. This initial production was attributed to γδ T cells and is followed two days later by the synthesis of IFN-γ by CD4^+^T cell [8].

Since IFN-γ is one of the most important molecules in modulating the acquired immune response, we investigated whether early IFN-γ production also occurs in mice immunized with the YF vaccine virus. We have used two different strains of mice (BALB/c and C57BL/6) based on their well-known differences in T_H_1/T_H_2 balance profiles as well as distinct capabilities of IFN-γ production [9–13]. We set out to demonstrate whether there was a correlation between the magnitude of early IFN-γ release and the quality of the acquired immune response. 

 In addition, YF 17D virus has been increasingly used as a vector for the delivery of antigens towards the development of new live attenuated vaccines. One important application of YF 17D virus as a vector is its use to develop HIV vaccines based on the fact that strong CD8^+^ T cell response is needed to control HIV or SIV infection. Accordingly, the YF 17D virus has been shown to elicit robust CD8^+^ T cell memory and effector responses [14]. In order to validate the use of 17D virus as a vector for HIV-SIV antigens, it is, therefore, important to define the features of the immune response elicited by the YF 17D virus and establish what should be retained by any recombinant thereof. In our previous work [8], performed in monkeys, we have compared the early IFN-γ production after immunization with nonrecombinant YF 17D-204 virus and recombinant 17D and Ad-5 carrying SIV genes, and we found that this phenomenon is a feature of 17D virus vaccination. In the present work, we studied the immune response of both mouse lineages after immunization with YF 17DD virus, one of the viral strains certified for human vaccination produced at Bio- Manguinhos/ FIOCRUZ, Brazil, and two different recombinant YF 17D viruses expressing a fragment of the Simian Immunodeficiency virus (SIV) Gag_45-269_ protein . It was confirmed that 17DD virus and 17D-204 recombinant derivatives expressing SIV antigens also trigger the early production of IFN-γ in vaccinated mice leading to significant CD8^+^ T cell response.

## Materials and Methods

### Cell Culture and Viruses

Vero cells were maintained in Earle’s 199 complete medium supplemented with 5% fetal bovine serum (FBS). Yellow Fever 17D vaccine (substrain YF 17DD) virus stock was generated after a single passage of the commercial YF 17DD vaccine virus (Bio-Manguinhos, RJ, Brazil) in VERO cells. We have used YF 17DD vaccine virus as a control in all experiments and the 17D recombinant viruses are all derived from 17D-204 because the backbone used came from 17D-204 virus. Whenever 17D recombinant viruses are cited it means 17D-204-derived viruses. The construction of recombinant viruses using 17D-204 backbone bearing an insertion of SIV virus Gag gene between the viral E and NS1 genes was described elsewhere [15,16]. Viable YF 17D virus was recovered (YF 17D/ SIV Gag _45-269_ ) and expressed a fragment of SIV Gag protein gene (residues 45 to 269) and was shown to be immunogenic in rhesus monkeys [15]. However, this gene segment contains an IRES element [17], that leads to the loss of the Gag insert during serial passages in VERO cell cultures. In order to overcome this limitation, we constructed a variant YF 17D virus in which the IRES element was knocked out, originating a more stable virus, called YF 17D/ SIV Gag Delta (∆) IRES, referred hereafter as YF 17D/ SIV Gag ∆ IRES [MG Santana, PC Neves and MC Bonaldo, unpublished].

### Animals

Female BALB/c mice or C57BL/6 mice, 4–6 weeks old were purchased from CEMIB-UNICAMP, Campinas, São Paulo and used in the YF 17D immunization studies. This study was carried out in strict accordance with the recommendations in the Guide for the National Council for Control of Animal Experimentation (CONCEA). The protocol was approved by the Committee on the Ethics of Animal Experiments (CEUA) of Oswaldo Cruz Foundation (Permit Number: L-47/2011). 

### Peptides

Measurement of specific CD8^+^ T cell responses elicited against YF 17DD was based on the use of peptides described as being immunodominant epitopes for each strain of mice. For BALB/c strain, the peptide used was CYNAVLTHV coming from the Envelope protein, positions 60-68 [18] and for C57BL/6 mice, the peptide was ATLTYRML from the NS3 protein, positions 268-275 [19].

To characterize the cellular response against the foreign protein, a set of 60 peptides of 15 amino acids overlapping by 11 (15 x 11), comprising the length between positions 45 and 269 of the first half of SIV mac239 Gag protein, was obtained from the AIDS Research and Reference Reagent Program, Division of AIDS, NIAID, NIH.

### Intracellular cytokine staining (ICCS)

In order to perform the kinetics studies, groups of 4 mice (both BALB/c and C57BL/6 in parallel), in each experiment, were subcutaneously injected with one dose in each side of dorsal area, with vehicle alone (Mock) or 100,000 PFU of YF 17D/ SIV Gag _45-269_, YF17D/ SIV Gag ∆ IRES or YF 17DD. After 1, 3, 5 and 7 days post-inoculation, one animal of each group was sacrificed and spleens as well as draining lymph nodes were recovered. The cells from both organs were isolated by standard methods and, in the case of spleens, red blood cells were lysed by using BD Lysis Solution (BD-Biosciences PharMingen, San Diego, CA). The leucocytes were then resuspended in culture medium RPMI 1640 supplemented with 1% HEPES 1M buffer, 2 mM L-Glutamine, 5 μM β-mercaptoethanol, 1 mM sodium pyruvate, 1% non-essential amino acid solution, 1% (V/V) vitamin (all from Invitrogen New Island, NY), 10% (V/V) Fetal Bovine Serum (Gibco- Invitrogen, New Island, NY) and 50 μg/mL gentamicin (Gibco- Invitrogen, New Island, NY). The cells were distributed in cluster tubes (1 X 10^6^/ tube) and incubated at 37° C, 5% CO_2_, for 6 hours with 10 μg/ml of brefeldin A (Sigma, St. Louis, MO) to prevent protein transport from the Golgi apparatus and the accumulation of cytokines *ex vivo*. After the incubation period, the cells were washed, stained for selected surface markers: CD3 PerCP (BD Pharmingen, San Diego, CA), CD8a PerCP (BD Pharmingen, San Diego, CA), CD4 Alexa 647 (BD Pharmingen, San Diego, CA), NK cells marker (DX-5) APC (eBiosciences, San Diego, CA ) or gamma-delta TCR APC (eBiosciences, San Diego, CA) and fixed overnight in 1% paraformaldehyde at 4°C. The following day the cells were permeabilized with 0.1% saponin in PBS containing 10 % FCS (Gibco- Invitrogen, New Island, NY) and stained for intracellular gamma interferon (IFN-γ PE, BD Pharmingen, San Diego, CA). After 60 minutes of staining, the cells were washed with PBS containing 10 % FCS and fixed in 1% paraformaldehyde for at least 30 minutes at 4°C.The cells were collected (100,000 inside the lymphocytes`gate) on Cyan ADP (7-color multiparametric flow cytometer) using Summit 4.3 software (Beckman Coulter, Brea, CA). The data were analyzed with FlowJo 7.6.5 for Windows software (Tree Star, Ashland, OR) and the values obtained were compared by using two-way ANOVA test, with Bonferroni post-test (GraphPad Prism 5.02 software). 

For specific responses assays, groups of 4 mice, in each experiment, were subcutaneously immunized with two doses, fifteen days apart, of vehicle alone or 100,000 PFU of YF17D/ SIV Gag _45-269_, YF17D/ SIV Gag Δ IRES or YF 17DD, in dorsal area. Splenocytes from immunized mice were recovered fourteen days after second dose and were assayed by ICCS for IL-2 and IFN-γ.The cell suspensions, obtained as described above, were in a final concentration of approximately 10^7^ cells/ml. All groups of harvested spleen cells (1 X 10^6^/ tube) were stimulated for 18-20 h with (1) medium alone, (2) whole YF 17DD inactivated virus (to verify CD4^+^ as well as CD8^+^ responses), (3) pool of peptides encompassing amino acids 45-269 of SIV Gag protein, (4) concanavalin A (2 μg/ml; Sigma, St. Louis, MO) in the presence of 1 μg of anti-CD28 and anti-CD49d monoclonal antibodies/ml (BD PharMingen, San Diego, CA). Five hours before the end of the incubation, 10 μg/ml of brefeldin A (Sigma, St. Louis, MO) was added to each tube. Cells were then washed (PBS 10% FCS) and stained for surface antigens with anti-CD8 PerCP and anti-CD4 Alexa 647 mouse monoclonal antibodies (BD-Biosciences PharMingen, San Diego, CA). The cells were washed again, fixed with paraformaldehyde 2%, permeabilized (PBS 10% FCS 0,1% Saponin) and stained with monoclonal antibodies anti-IL-2 FITC and anti-IFN-γ PE (BD-Biosciences PharMingen, San Diego, CA) followed by multiparametric flow cytometry on Cyan ADP 7-color flow cytometer using Summit software (BD Biosciences, San Jose, CA). The data were analyzed with FlowJo 7.6.5 for Windows software (Tree Star, Ashland, OR) and the values obtained were compared by using Mann-Whitney T test (GraphPad Prism 5.02 software). 

### IFN-γ ELISpot assay

For the IFN-γ ELISpot assay, groups of 4 or 5 mice, in each experiment, were immunized as described above (ICCS section). Fourteen days after second immunization, the mice were sacrificed and the spleens removed. Splenocytes were isolated by standard methods and red blood cells lysed by using BD Lysis Solution (BD-Biosciences PharMingen, San Diego, CA). The leucocytes were then ressuspended in culture medium RPMI 1640 supplemented with 1 M HEPES buffer, 2 mM L-Glutamine, 5 μM β-mercaptoethanol, 1 mM sodium pyruvate, 1% non-essential amino acid solution, 1% (V/V) vitamin (all from Invitrogen New Island, NY), 10% (V/V) Fetal Bovine Serum (Gibco- Invitrogen, New Island, NY) and 50 μg/mL gentamicin (Gibco- Invitrogen, New Island, NY). The suspensions were then distributed in previously prepared IFN-γ ELISpot plates.

IFN-γ ELISpot plates were prepared using IFN-γ ELISpot pre-coated plates kit (Mabtech), according to the manufacture’s protocol. Briefly, pre-coated ELISpot plates were blocked with supplemented RPMI 1640 for at least 2 hours at 37°C and 2 X 10^5^ cells/ well of each mouse were added. The cells were cultured in the presence of medium alone (no stimulus), 2 μg/mL concanavalin A, 2 μg/mL of YF 17D specific peptides or 5 mM of SIV Gag peptide pool. After 20 hours of culture, the plates were washed and incubated with biotinylated anti- IFN-γ for 2 hours at room temperature followed by ALP-conjugated Streptavidin for 1 hour at room temperature. The signal was developed with NBT/BCIP substrate available with the kit. Finally, the spots on plate membranes were counted using an ImmunoSpot image analyser (CTL, Cleveland, OH, USA). The results are presented after subtraction of the background and compared using Mann-Whitney test (GraphPad Prism 5.02 Program). The differences were considered significant when *P* < 0.05.

### Plaque Reduction Neutralization Test 50 (PRNT_50_)

 Groups of 4 or 5 mice, in each experiment, from both BALB/c and C57BL/6 strains, were subcutaneously immunized with two doses, fifteen days apart, of vehicle alone or 100,000 PFU of YF17D/ SIV Gag _45-269_, YF17D/ SIV Gag Δ IRES or YF 17DD. Two weeks after the last immunization, mice were bled by using cardiac puncture. Serum samples were treated for 30 minutes at 56°C and stored at -20°C. YF neutralizing antibody titer was determined by plaque reduction neutralization test with 50% endpoint (PRNT_50_) in 96-well plates with Vero cells as described previously [20]. The values of neutralizing antibody titers of each experimental group were compared using Mann-Whitney test (GraphPad Prism 5.02 Program). The differences were considered significant when P < 0.05.

### ELISA assay

Microplates (Maxisorb; Nunc, USA) were coated with 10 μg/ mL of whole virus particle obtained in cell culture (YF 17DD vaccine, first passage in VERO cells) diluted in 100 μL of carbonate buffer 0.05M pH 9.6 and incubated overnight at 4° C. The following day , plates were washed three times with phosphate-buffered saline containing 0.05% (v/v) of Tween-20 (Wash Solution) and blocked at 37°C for two hours with blocking solution (PBS containing 5% (w/v) non-fat milk with 1% (w/v) of bovine serum albumin (BSA, Sigma Co.). The serum samples were obtained as described in neutralizing antibody assay, diluted 1/200 in blocking solution and distributed, in duplicate wells, for total IgG dosage. An anti-YF virus antibody (clone OG5, ABCAM) was employed to derive a standard curve, in the range of 5 to 0.039 μg/mL. The standard curve and samples were incubated at room temperature (RT) for 1 hour and unbound antibodies were washed away with wash solution. Anti-IgG conjugated to HRP (Southern Biotech, Birmingham-AL) diluted 1/2,000 in blocking solution, 100μL/ well, was added. After one-hour incubation at room temperature, the excess labeled antibody was removed by washing five times with wash solution, and the reaction was developed with tetramethylbenzidine substrate (Sigma, USA). After 15 min, the reaction was stopped by adding 2 M H_2_SO_4_ solution and the plates were read at 450 nm on VERSAmax ELISA reader (Molecular Devices). A similar protocol was used to detect IgG1 and IgG2a isotypes. In this case, after serum incubation (1/50, in duplicates), each half part of the plate received antimouse-IgG1 conjugated to HRP or anti-IgG2a conjugated to HRP (Southern Biotech, Birmingham-AL), so that each serum sample was measured for a given isotype at the same time and in the same plate. The results are expressed in μg/ mL for total IgG and compared using Mann-Whitney test (GraphPad Prism 5.02 Program). The cut-off was calculated as the mean of concentrations found for vehicle immunized mice sera ± 2 SD and was defined as 5.59 μg/mL. The differences were considered significant when P < 0.05. For IgG1 and IgG2a isotypes, the results were expressed as a ratio of IgG2a/ IgG1 optical densities (ODs) (representing the T_H_1/T_H_2 balance) for each mouse. The ODs for vehicle immunized mice were found to be consistently lower than 0.100.

## Results

In order to determine if there was early IFN-γ production in mice after immunization with YF 17DD and recombinant viruses as we had previously observed in rhesus monkeys and humans [7,8] we vaccinated two different mouse strains, C57BL/6 and BALB/c, with a single dose of vehicle alone (medium 199), YF 17DD, YF17D SIV/ Gag _45-269_ or YF17D/ SIV Gag Δ IRES viruses. These two lineages were chosen because they were shown to have different capacities of promoting early IFN-γ production after microbial challenge [9,10]. We hypothesized that these strains could display distinct abilities regarding the promotion of early IFN-γ synthesis as well as eliciting of a specific immune response against YF viruses. After 1, 3, 5 and 7 days post-inoculation (p.i.), one animal from each group was sacrificed and spleens as well as both draining lymph nodes were recovered and analyzed for ex vivo IFN-γ generation and cell marker expression, in three different experiments. The gating strategies are explained in figures S1 and S2.

### Production of IFN-γ by γδ T cells in lymph nodes and spleen of mice immunized with YF 17DD and recombinant viruses

**Figure 1:**
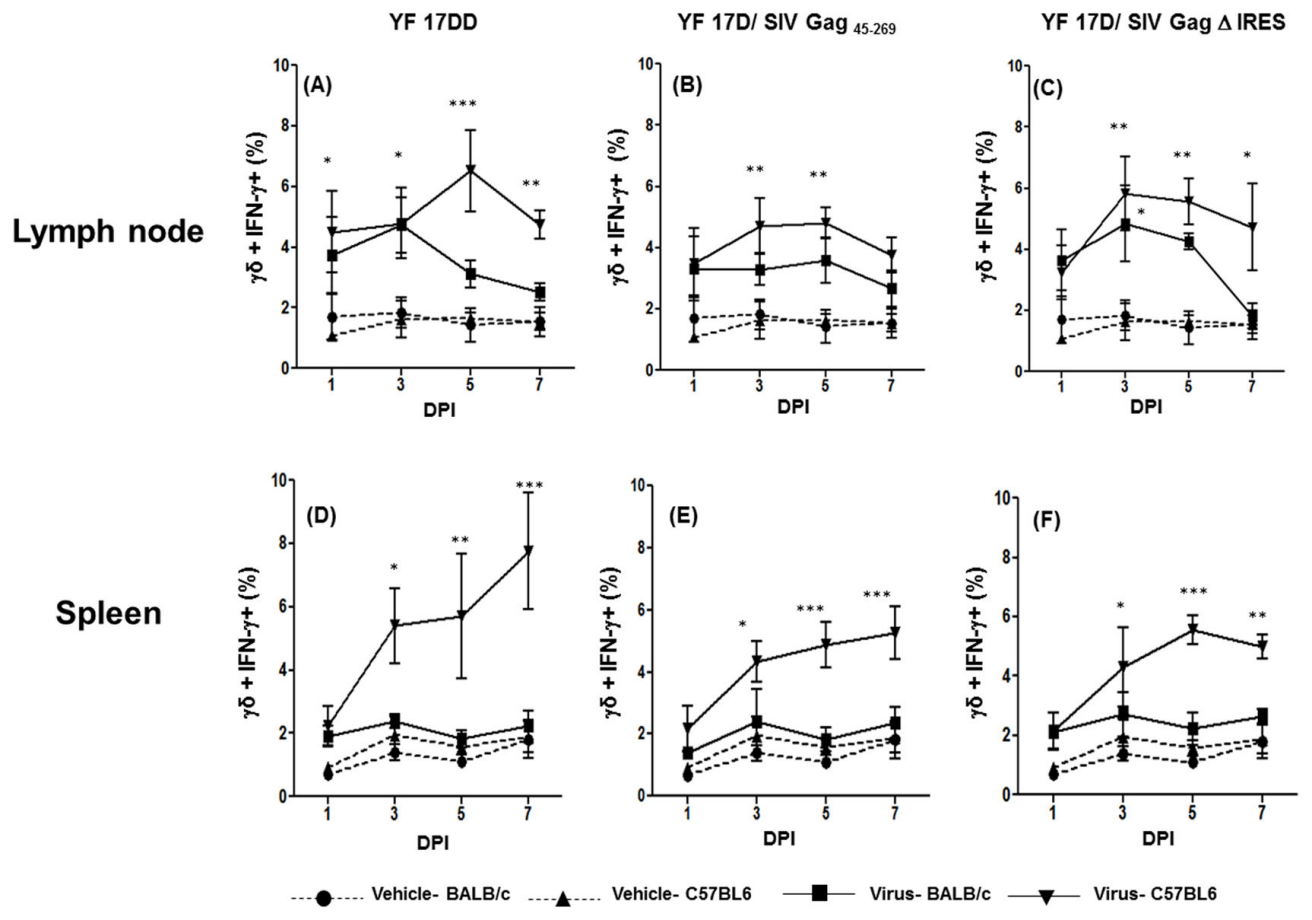
Production of IFN-γ by γδ T cells in the first week after 17DD and 17D/ SIV Gag recombinants immunization in mice. Percentages of γδ^+^ T cells+ IFN- γ^+^ cells in draining lymph nodes and spleens of mice following 17 DD – (Lymph node-panel A; Spleen- panel D), 17D SIV Gag _45-269_ (Lymph node-panel B; Spleen- panel E) and 17D Δ IRES (Lymph node- panel C, Spleen- panel F) immunization in C57BL/6 and BALB/c mice. Statistical analysis was performed by Two- Way ANOVA with Bonferroni post-test. * *p*<0.05, ** *p*<0.01, *** *p*<0.001. The means are representative of three independent experiments. N= 3 animals/ experimental point.

In our previous study using rhesus monkeys, we determined that γδ T lymphocytes were the first cells to produce IFN-γ after YF 17D immunization [8]. Accordingly, on the first day post-inoculation (d.p.i.), a significantly higher number of γδ^+^IFN-γ^+^ cells was observed in relation to the vehicle alone in the lymph nodes of C57BL/6 mice immunized with the vaccine YF 17DD virus (Figure 1A). At 3 d.p.i, the number of γδ+ IFN-γ+ cells increased, peaking at 5 d.p.i., followed by a drop at 7 d.p.i.. In BALB/c lymph nodes we observed also γδ^+^ IFN-γ^+^ cells early in the first week p.i. Despite the fact that the percentage of γδ^+^ IFN-γ^+^ cells was lower for BALB/c mice immunized with the same virus in all time points, it was not statistically different from vehicle injected mice and significantly lower (*p*<0.05) than C57BL/6 mice at 5 d.p.i.

Similar results were observed for recombinant viruses, but a delay in the appearance of γδ^+^ IFN-γ^+^ lymph node cells was observed. Significant differences between vehicle and viruses emerged only on day three after immunization. For YF 17D/ SIV Gag _45-269_, in C57BL/6 mice, higher numbers of γδ^+^ IFN-γ^+^ cells persisted until day 5 (Figure 1B). For YF 17D/ SIV Gag Δ IRES, those cells also appeared on day three and persisted until day seven in C57BL/6 mice (Figure 1C). With regards to BALB/c strain, the detection of γδ^+^IFN-γ^+^ cells was also delayed with significant differences from the control group observed only on day three after immunization with YF 17D/ SIV Gag Δ IRES and being significantly lower (*p*<0.05) than C57BL/6 immunized group on day 7. In addition, the γδ T cells of lymph nodes from C57BL/6 mice inoculated with any of the YF 17D viruses yielded a more sustainable IFN-γ response as compared to γδ T cells from BALB/c mice . 

 In the spleen of C57BL/6 mice significant numbers of γδ^+^ IFN-γ^+^ cells increased on day 3 after immunization with all viruses (Figure 1D, E and F). In this organ, the differences between the numbers of γδ^+^ IFN-γ^+^ cells in C57BL/6 and BALB/c mice are more pronounced, being significantly lower in the latter, on days 5 and 7 for YF 17DD virus (Figure 1D), YF 17D/ SIV Gag_45-269_ (Figure 1E) and for YF 17D/ SIV Gag Δ IRES (Figure 1F). 

### Production of IFN-γ by NK cells in lymph nodes and spleen of mice immunized with YF 17DD and recombinant viruses

**Figure 2:**
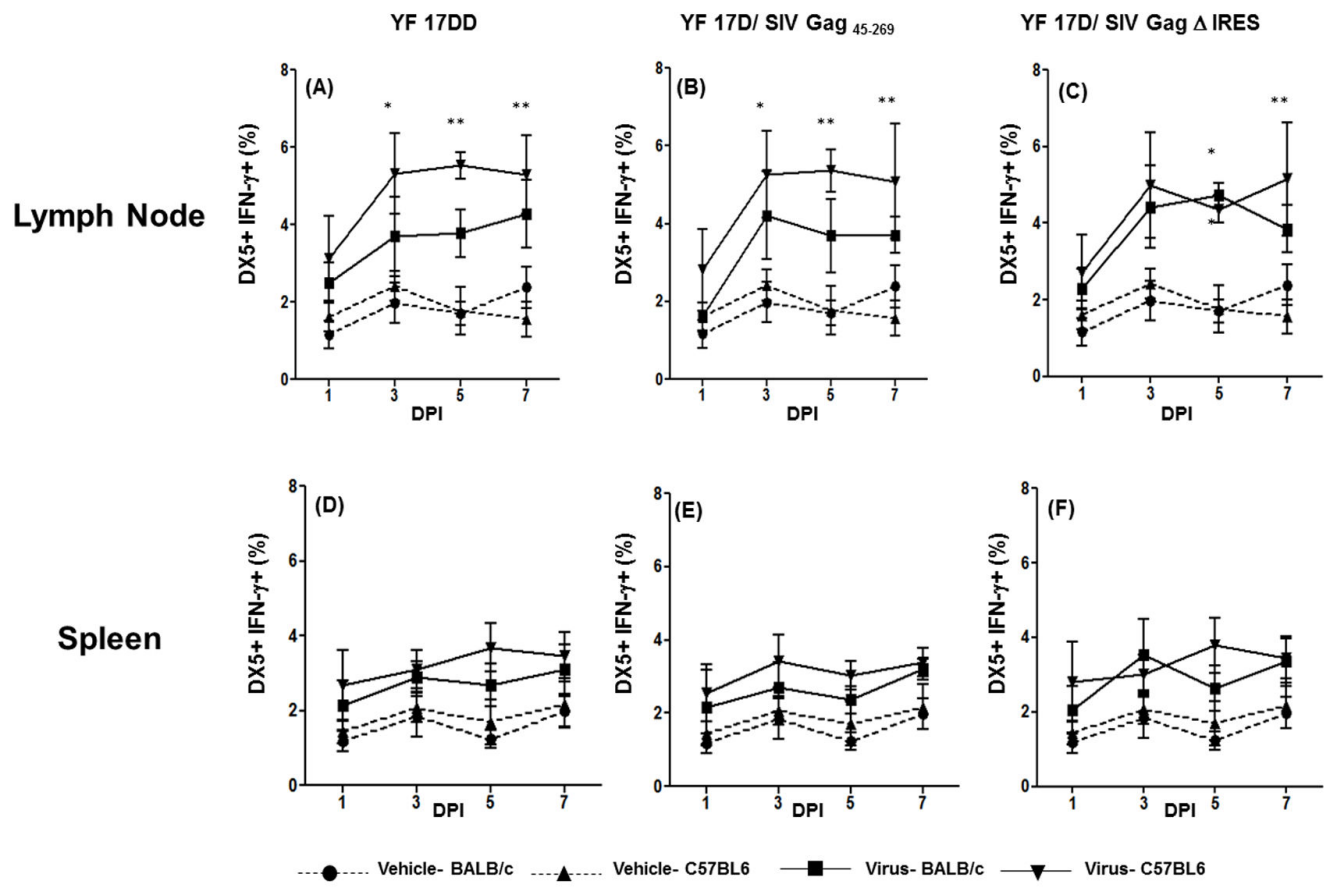
Production of IFN-γ by NK cells in the first week after 17DD and 17D/ SIV Gag recombinants immunization in mice. Percentages of DX5^+^ IFN-γ^+^ cells in draining lymph nodes and spleens of mice following 17 DD – (Lymph node-panel A; Spleen- panel D), 17D SIV Gag _45-269_ (Lymph node-panel B; Spleen- panel E) and 17D Δ IRES (Lymph node- panel C, Spleen- panel F) immunization in C57BL/6 and BALB/c mice. Statistical analysis was performed by Two- Way ANOVA with Bonferroni post-test. * *p*<0.05, ** *p*<0.01, *** *p*<0.001. The means are representative of three independent experiments. N= 3 animals/ experimental point.

 NK cells are considered one of the most important IFN-γ-secreting cells induced in the initial host immune responses in several infections [21]. In fact, in lymph nodes, it was possible to see a significant increase of DX5+ (mouse NK cell marker) IFN-γ^+^ cells since day 3 after immunization with all three viruses (Figure 2 A, 2B and 2C). Despite the fact that the numbers of DX5^+^IFN-γ^+^ cells are, in general, higher after C57BL/6 immunization (those are significantly different from mock injected mice), we could observe an increase in DX5^+^IFN-γ^+^ cells also in BALB/c immunized mice. This increase was statistically different from mock injected mice only on day 5 after YF 17D/ SIV Gag Δ IRES injection (Figure 2C). In the spleen, we could not demonstrate any differences among viruses and mock injected groups (Figure 2 D, 2 E and 2 F). 

### Production of IFN-γ by T CD4^+^ cells in lymph nodes and spleen of mice immunized with YF 17DD and recombinant viruses

**Figure 3:**
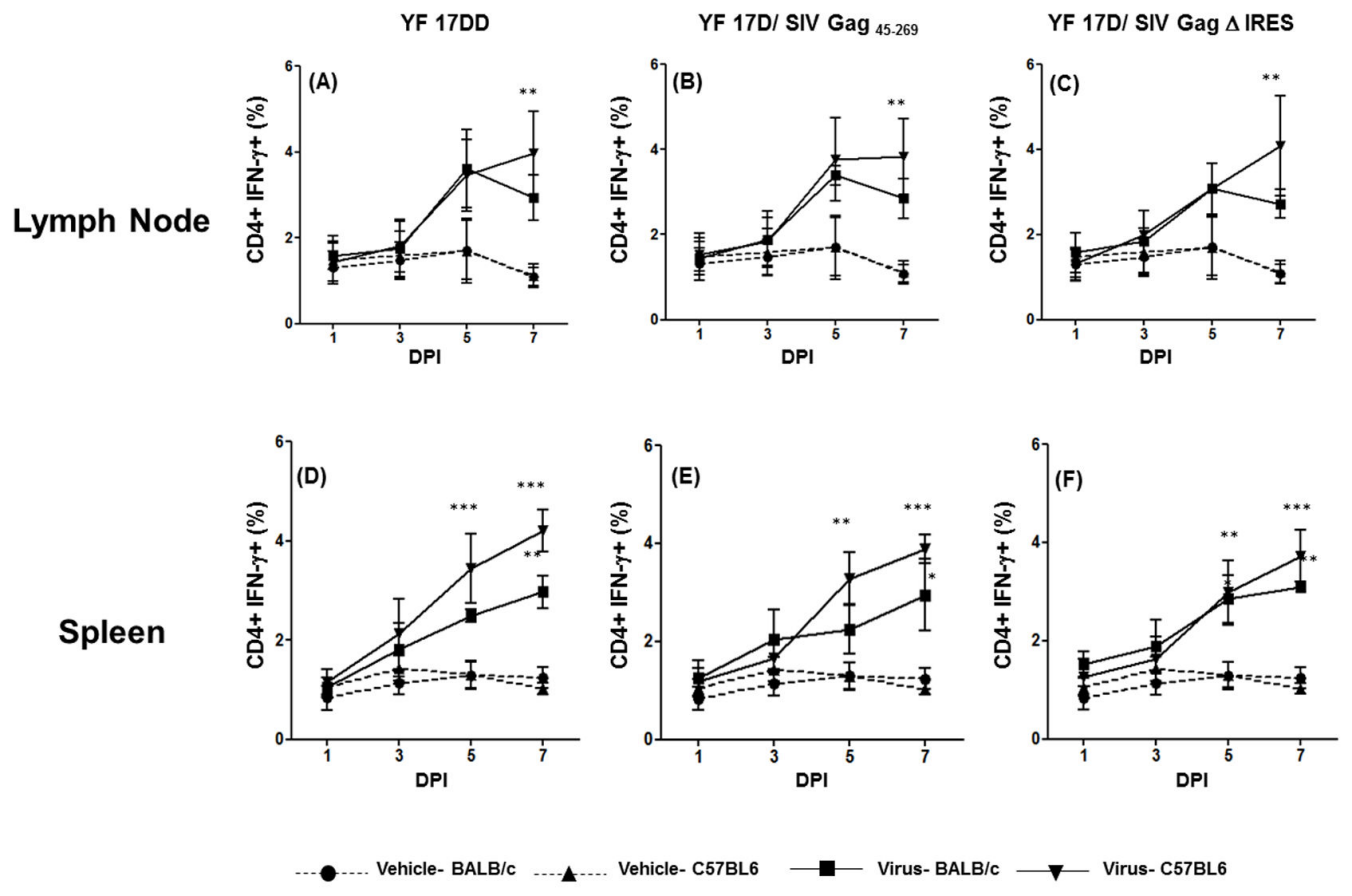
Production of IFN-γ by CD4^+^ T cells in the first week after 17DD and 17D/ SIV Gag recombinants immunization in mice. Percentages of CD4^+^ IFN- γ^+^ cells in draining lymph nodes and spleens of mice following 17 DD – (Lymph node-panel A; Spleen- panel D), 17D SIV Gag _45-269_ (Lymph node-panel B; Spleen- panel E) and 17D Δ IRES (Lymph node- panel C, Spleen- panel F) immunization in C57BL/6 and BALB/c mice. Statistical analysis was performed by Two- Way ANOVA with Bonferroni post-test. * *p*<0.05, ** *p*<0.01, *** *p*<0.001. The means are representative of three independent experiments. N= 3 animals/ experimental point.

 It is well known that the release of IFN-γ by innate immune cells may influence dendritic cell maturation and consequently CD4^+^ T cells polarization to T_H_1 lineage [22,23]. In our previous study in rhesus monkeys, IFN-γ production by γδ T cells is followed by the emergence of T CD4^+^IFN-γ^+^ cells in peripheral blood [8]. In the present study, we could detect an increase of CD4^+^IFN-γ^+^ cells both in lymph nodes and spleens of both mouse lineages immunized with each of the 3 viruses. This increase is statistically significant in the lymph nodes of C57BL/6 mice by day 7 after immunization (YF 17DD- Figure 3 panel A; YF 17D/ SIV Gag _45-269_- Figure 3 panel B and YF 17D/ SIV Gag Δ IRES – Figure 3 panel C). In BALB/c mice, the differences between virus and vehicle-immunized animals were not statistically significant, despite the observed increase in these cells from virus-immunized groups.

In the spleen, the rise in CD4^+^IFN-γ^+^ cells is statistically significant from day 5 in C57BL/6-immunized mice (Figure 3 YF 17DD- panel D; YF 17D/ SIV Gag _45-269_- panel E and YF 17D/ SIV Gag Δ IRES – panel F) and is even higher by day 7 after immunization. For BALB/c mice, significant differences were only detected on day 7 after immunization.

### Production of IFN-γ by T CD8^+^ cells in lymph nodes and spleen of mice immunized with YF 17DD and recombinant viruses

**Figure 4:**
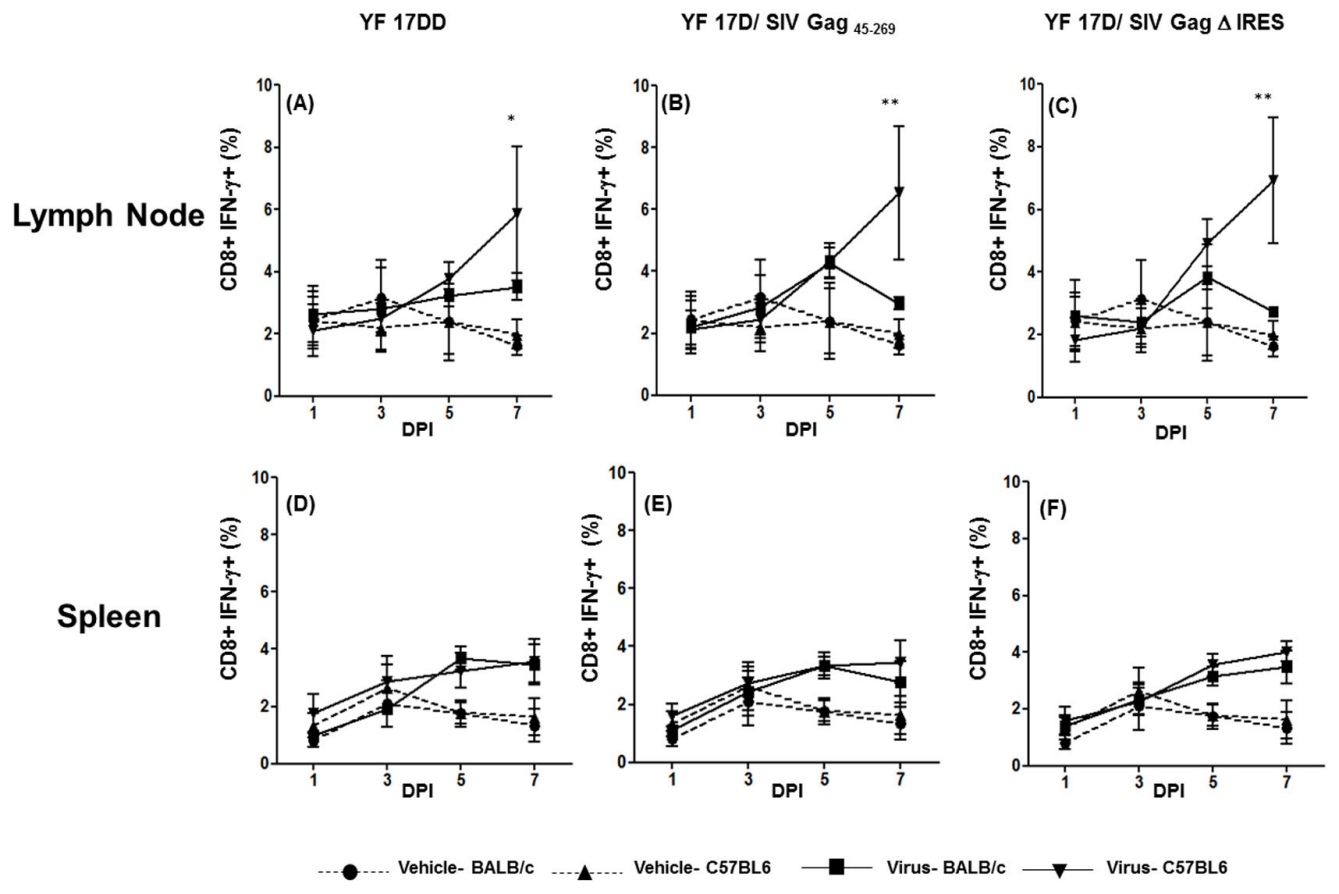
Production of IFN-γ by CD8^+^ T cells in the first week after 17DD and 17D/ SIV Gag recombinants immunization in mice. Percentages of CD8^+^ IFN- γ^+^ cells in draining lymph nodes and spleens of mice following 17 DD – (Lymph node-panel A; Spleen- panel D), 17D SIV Gag _45-269_ (Lymph node-panel B; Spleen- panel E) and 17D Δ IRES (Lymph node- panel C, Spleen- panel F) immunization in C57BL/6 and BALB/c mice. Statistical analysis was performed by Two- Way ANOVA with Bonferroni post-test. * *p*<0.05, ** *p*<0.01, *** *p*<0.001. The means are representative of three independent experiments. N= 3 animals/ experimental point.

 The CD8^+^ T cells are key players in the immune response during viral infections, since after activation; they are capable of eliminating infected cells by specific mechanisms. It has also been shown that CD4^+^ T cells can shape the magnitude as well as the quality of primary and/or secondary CD8^+^T-cell responses [24]. It is noteworthy that, in this study, we were able to demonstrate an increase in CD8^+^IFN-γ^+^ cells immediately after the appearance of CD4^+^IFN-γ^+^ cells in lymph nodes, but only in C57BL/6 immunized mice (Figure 4 panels A, B and C). 

In the spleen, although we observed a slight rise in CD8^+^IFN-γ^+^ cells in both mouse lineages by day 7 after immunization, these differences were not statistically significant as compared to the control group (Figure 4 panels D, E and F). We found that YF 17DD and recombinant viruses are able to induce IFN-γ production early after mice infection in both lineages. The early IFN-γ production reached higher levels in C57BL/6 mice compared to BALB/c mice, mainly in relation to NK cells in lymph nodes and to γδ T cells in both secondary lymphoid organs. The next step of our study was to investigate whether these differences observed in early IFN-γ production would be reflected in the specific responses developed against the viruses.

### Specific cellular responses in C57BL/6 and BALB/c mice after immunization with YF 17DD and recombinant viruses

 IFN-γ is one of the most important molecules regulating cellular immune response. It has been demonstrated that IFN-γ produced by innate immune cells may influence dendritic cells (DC) maturation and consequently affect the quality of specific cell immunity [25–27]. 

We demonstrated above that the magnitude of the early IFN-γ response is higher in C57BL/6 mice in comparison to BALB/c mice. We then investigated whether these differences could influence different specific adaptive immune responses. We first performed an analysis of T cell specific response in these two different mouse lineages. For this purpose, groups of mice were subcutaneously immunized with two doses, fifteen days apart, of vehicle alone or virus. Fifteen days after the last immunization, spleen cells were recovered and cultured with inactivated 17DD virus and Gag peptide pools. The gating strategies and representative figures are illustrated in Figure S3.

**Figure 5:**
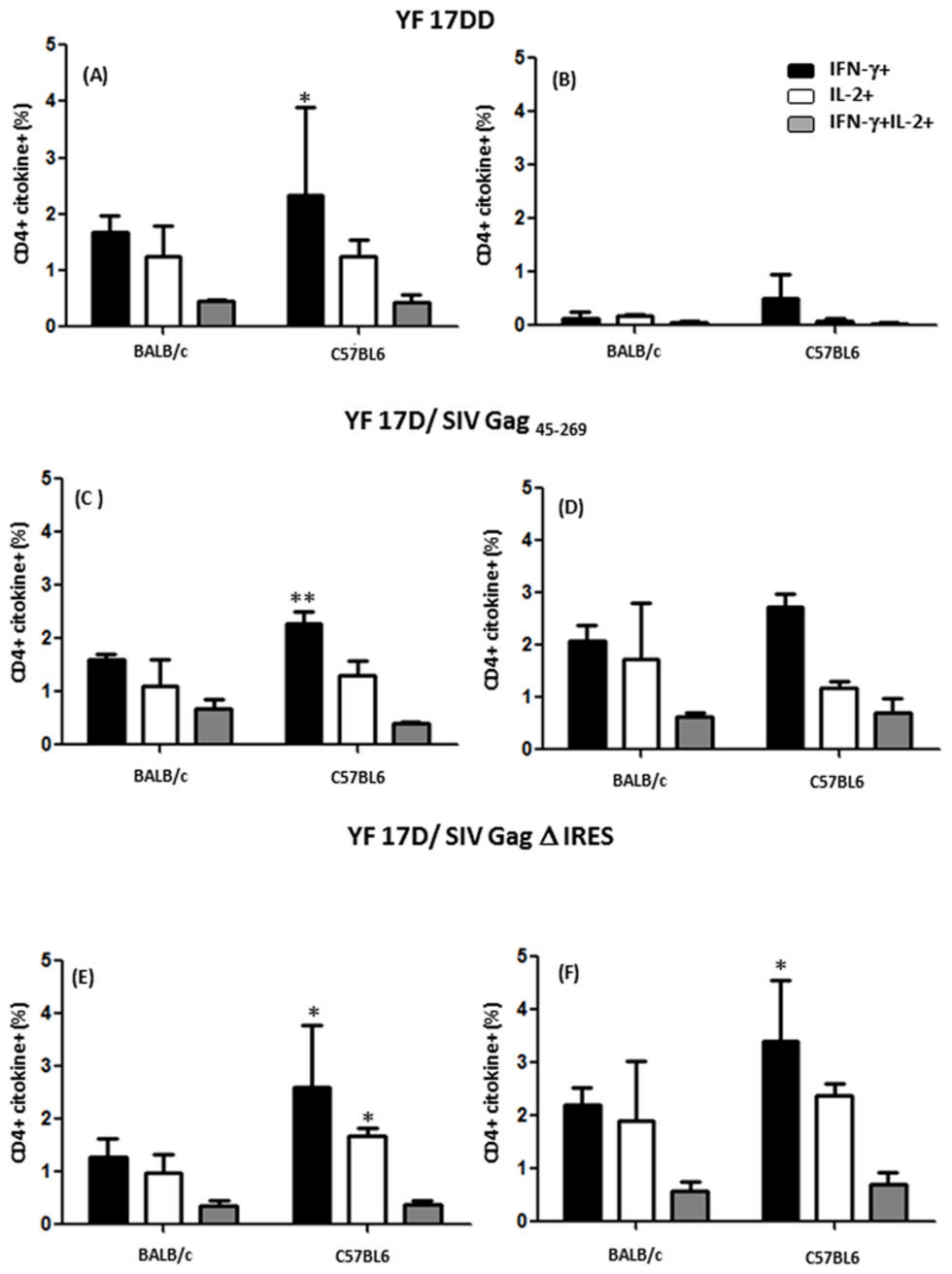
Functional profile of CD4^+^ T cell responses in mice immunized with 17DD and 17D/ SIV Gag recombinants. ICCS for YF-specific and Gag-specific CD4^+^ T-cells at day 14 after immunization on secretion of IFN-γ and/or IL-2 following stimuli with antigen (whole inactivated YF 17DD virus -panels A, C and E and peptide mixtures spanning amino acids 1–291 of Gag -panels B, D and F). Bar graphs indicate the mean frequency of CD4^+^ splenocytes specific to YF and Gag capable of producing IFN-γ (black), IL-2 (white) or both (grey). Error bars represent the standard error of the mean. Differences between cytokine + cells were statistically significant between BALB/c and C57BL/6 mice (* p<0.05, **p<0.01). The means are representative of three independent experiments. N= 4 animals/ experiment.

**Figure 6:**
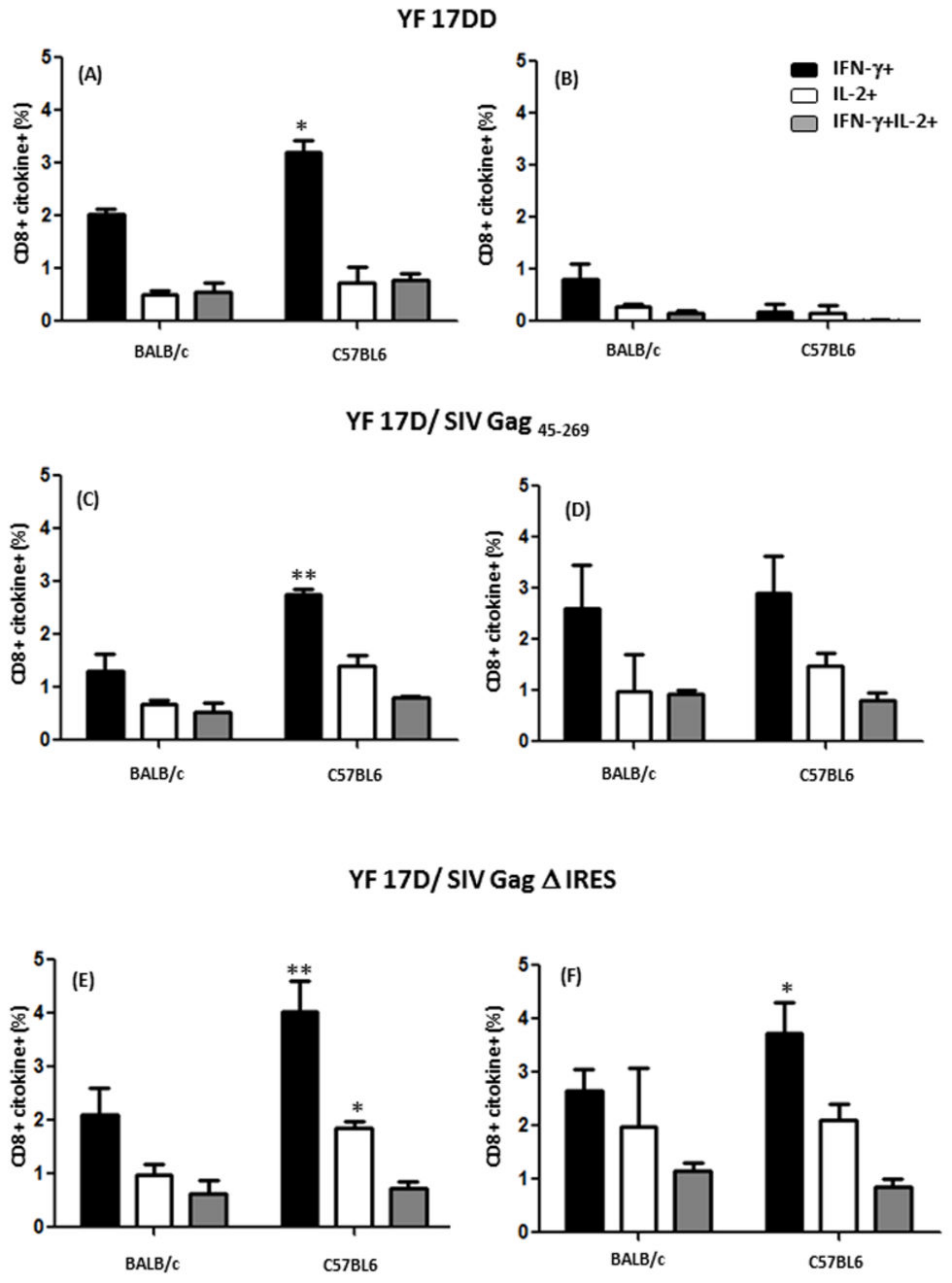
Functional profile of CD8^+^ T cell responses in mice immunized with 17DD and 17D/ SIV Gag recombinants. ICCS for YF-specific and Gag-specific CD8^+^ T-cells at day 14 after immunization on secretion of IFN-γ and/or IL-2 following stimuli with antigen (whole inactivated YF 17DD virus -panels A, C and E and peptide mixtures spanning amino acids 1–291 of Gag -panels B, D and F). Bar graphs indicate the mean frequency of CD8^+^ splenocytes specific to YF and Gag capable of producing IFN-γ (black), IL-2 (white) or both (grey). Error bars represent the standard error of the mean. Differences between cytokine + cells were statistically significant between BALB/c and C57BL/6 mice (* p<0.05, **p<0.01). The means are representative of three independent experiments. N= 4 animals/ experiment.

 As shown in figures 5 and 6, significant statistical differences in specific T cell responses against YF 17DD virus antigen could be found for CD4^+^ (Figure 5 panels A, C and E) as well as CD8^+^ (Figure 6 panels A, C and E) only for IFN-γ- producing cells, with a higher percentage of these cells occurring in C57BL/6 mice immunized with all three viruses as compared to BALB/c mice. This observation points to the possibility that early IFN-γ may be influencing the development of effector memory cells. For SIV Gag antigens, CD4^+^ (Figure 5 panels B, D and F) and CD8^+^ (Figure 5 panels B, D and F) responses were better in C57BL/6 in comparison to BALB/c mice only for YF 17D/ SIV Gag Δ IRES, with the appearance of effector memory cells as well as central memory cells (IL-2+). As expected, we could not see any responses against Gag antigens in splenocytes from YF17DD immunized mice (Figure 5 panel B and Figure 6 panel B).

### Magnitude of CD8^+^ T cell responses in C57BL/6 and BALB/c mice after immunization with YF 17DD and recombinant viruses

**Figure 7:**
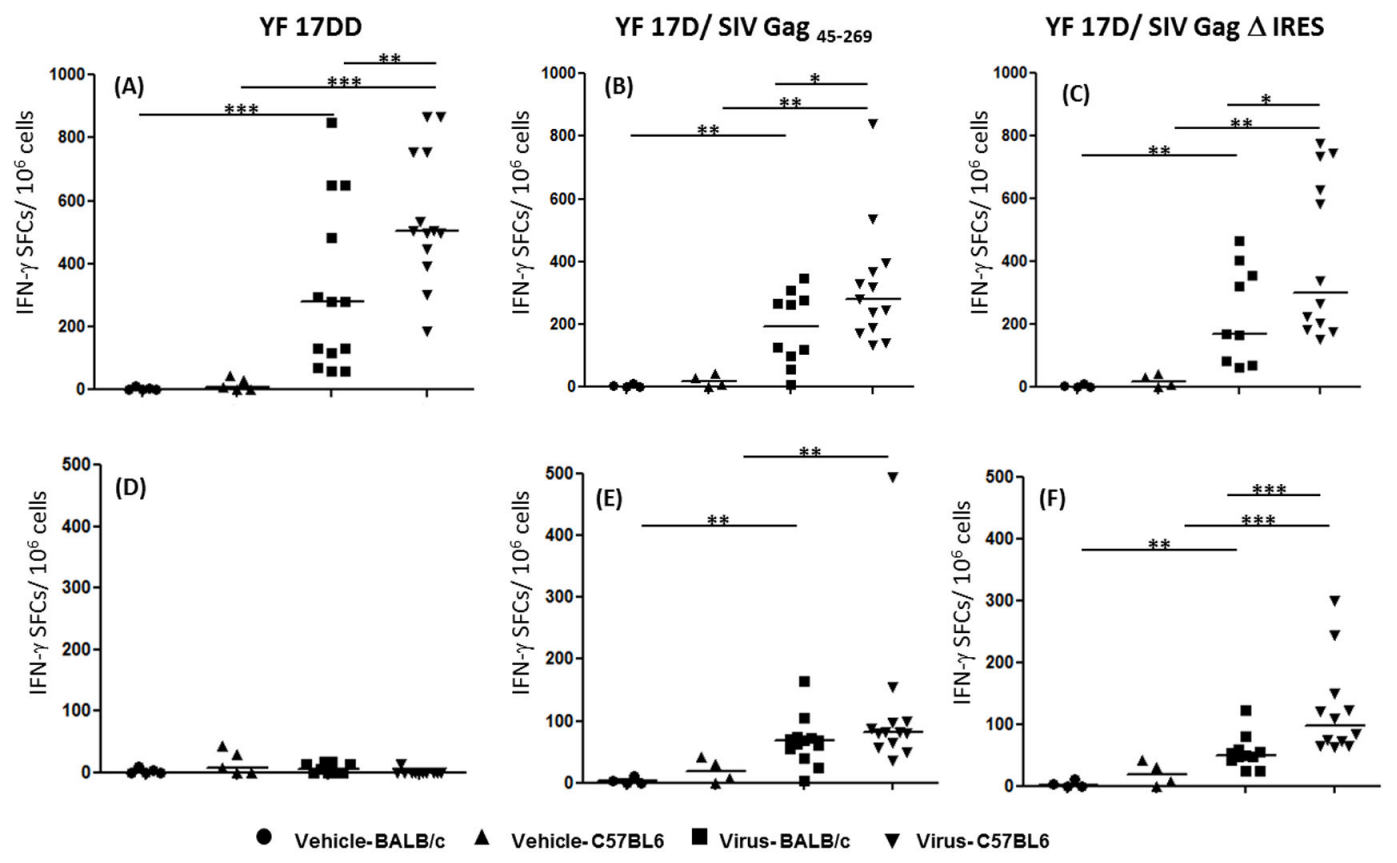
Magnitude of YF and Gag-specific CD8^+^ T-cell responses in animals vaccinated with YF 17DD and YF 17D recombinant viruses. Groups of mice (C57BL/6 strain and BALB/c strain) were immunized twice 15 days apart with vehicle medium (mock), YF vaccine (17 DD), recombinant 17 D SIV-Gag _45-269_ or recombinant 17 D Δ IRES virus. Spleen cells of each mouse were recovered 14 days after second immunization and cultured with 2 μg/mL of YF CD8 specific peptides (Panel A-C) or 5 mM of SIV Gag peptide pool (Panel D-F). Results are representative of three independent experiments and are normalized to non-stimulated cells control. Bars represent the medians. Statistics were done by Mann-Whitney T test. * *p*<0.05, ** *p*<0.01, *** *p*<0.001.

In order to confirm the possible influence of early IFN-γ production on the magnitude of specific CD8^+^ T cell response, we have performed ELISpot assays after immunization with the three viruses in each mouse strain. The splenocytes were recovered as explained above and cultured with specific immunodominant peptides of YF 17D virus (CYNAVLTHV for BALB/c mice and ATLTYRML for C57BL/6 mice) or with a pool of peptides of SIV Gag protein. As expected, the number of IFN- γ spot forming colonies (SFCs) in response to YF antigens is significantly higher in C57BL/6 mice when compared to BALB/c mice after immunization with the human vaccine 17DD virus (Figure 7, panel A). The same pattern is observed for recombinant viruses (Figure 7, panels B and C). In relation to Gag antigen, in accordance with ICCS assay data, the differences between C57BL/6 mice and BALB/c mice were also significant only after immunization with YF 17D/ SIV Gag Δ IRES virus (Figure 7 panel F) but not YF17D/ SIV Gag _45-269_ (Figure 7, panel E). As seen before, splenocytes from animals immunized with YF 17DD vaccine virus did not respond to stimulus with Gag antigen (Figure 7, panel D). 

### Specific antibody responses in C57BL/6 and BALB/c mice after immunization with YF 17DD and recombinant viruses

 Neutralizing antibodies are thought to be the major correlate of protection against yellow fever [28]. Emerging evidences point to the pivotal role of innate immunity in controlling the magnitude of antibody response [29]. Hence, in this work, we have also investigated antibody response after immunization with YF attenuated vaccine virus and its recombinant derivatives in both mouse lineages. 

**Figure 8:**
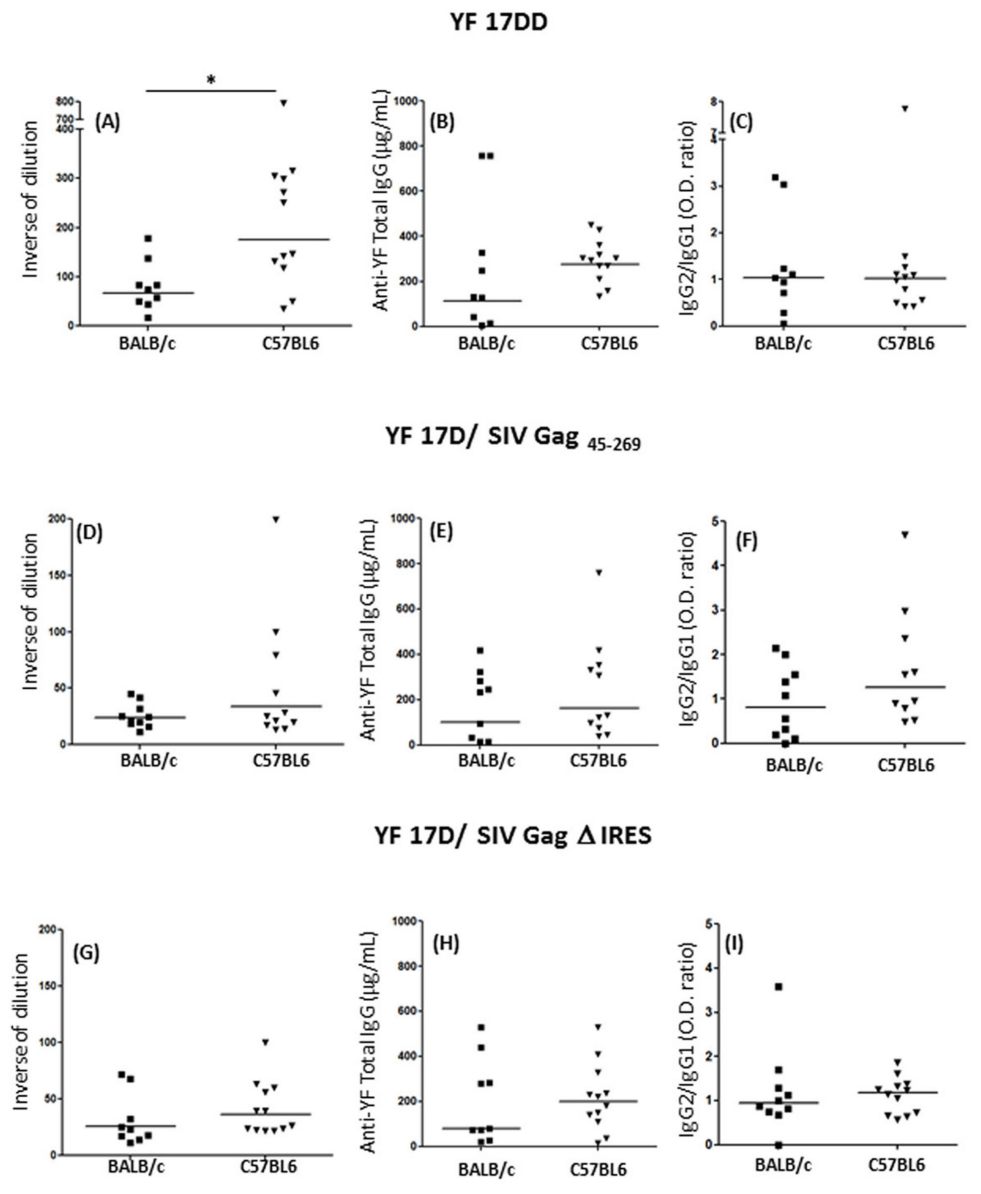
Humoral immune responses in animals vaccinated with YF 17DD and YF 17D recombinant viruses. Neutralizing antibodies levels, Total YF- IgG levels and IgG2a/ IgG1 ratios in mice sera following YF 17 DD (panels A - C), 17D SIV Gag _45-269_ (panels D - F) and 17D Δ IRES (panels G - I) virus immunization in C57BL/6 and BALB/c mice. Statistical analysis was performed by Mann-Whitney T test. * *p*<0.05, ** *p*<0.01, *** *p*<0.001. The medians are representative of three independent experiments.

As a first approach, we have measured neutralizing antibodies using a classical method of plaque reduction neutralization test (PRNT_50_). As shown in Figure 8 panel A, neutralizing antibody titers in C57BL/6 mice were significantly higher than in BALB/c mice fifteen days after second immunization with YF17DD virus. However, we could not observe the same profile for the recombinant YF 17D viruses, for which lower titers of neutralizing antibodies were seen in both mouse lineages (Figure 8 panels D and G). This profile was expected since 17D viruses with insertions between E and NS1 viruses are, in general, more attenuated than the commercial non-recombinant vaccine virus [32]. It is noteworthy that the analysis of anti-YF 17D total IgG revealed a tendency to higher titers of antibodies for all three viruses in C57BL/6 mice (Figure 8, panels B, E and H), although these differences were not significant as compared to mean titers reached in BALB/c. Nevertheless, antibodies exert their antiviral effects not only by virtue of the capacity to bind viruses and directly neutralize infectivity in a stoichiometric way, but also via effector functions coordinated by the crystalizable fragment (Fc) region of the antibody heavy chain [30,31]. In this aspect, IFN-γ exerts an essential role during class switch, leading to the production of more adequate class of antibody against viral infections [32,33]. Thus, we have decided to ascertain the possibility of existing differences in IgG isotypes produced against yellow fever E protein after immunization of both mouse lineages with the three viruses, since these lineages differ in their ability to produce IFN-γ. 

**Figure 9:**
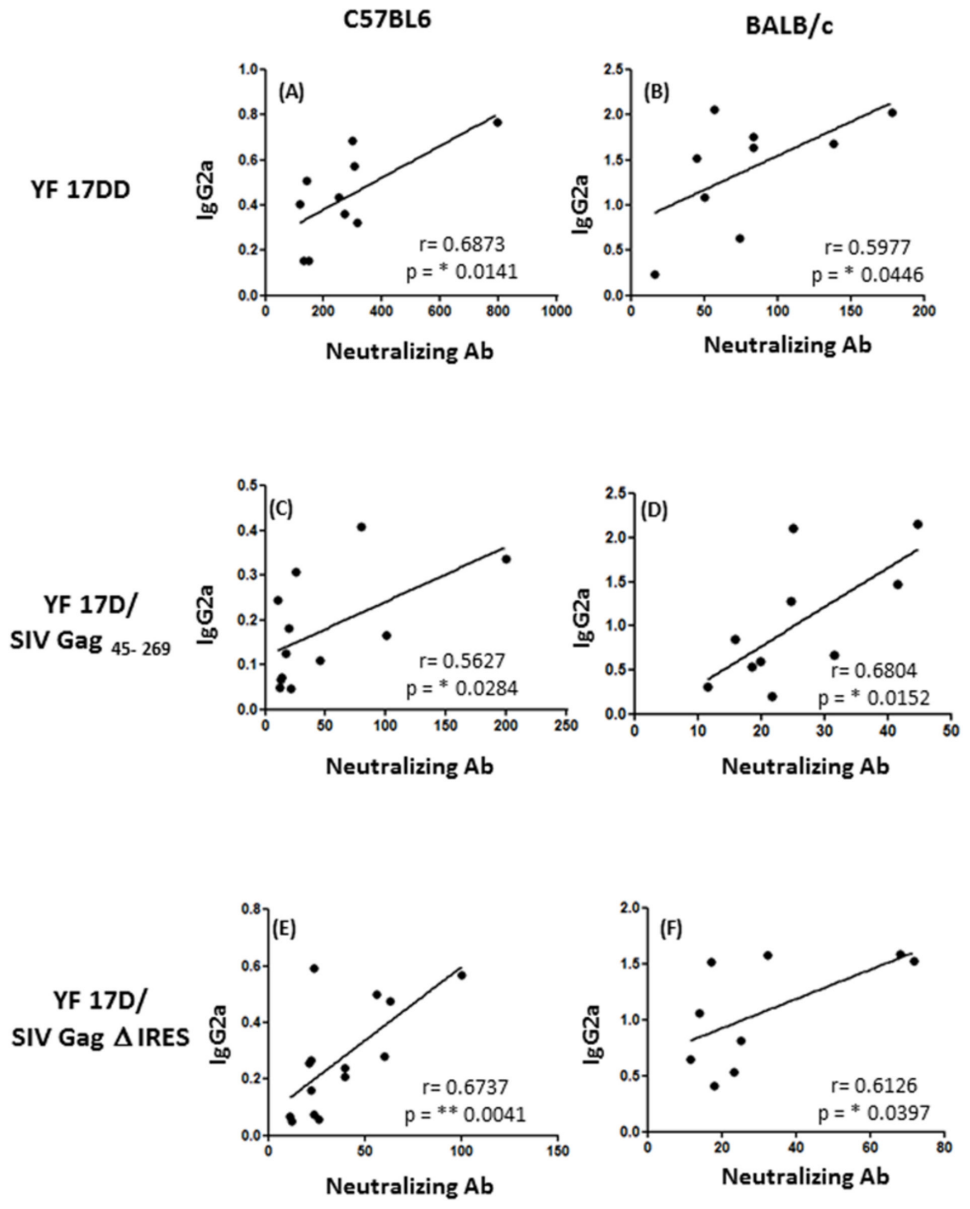
Correlations between neutralizing antibodies and IgG2a levels in sera of mice immunized with YF 17DD and YF 17D recombinant viruses. Neutralizing antibodies levels and IgG2a O.Ds in mouse sera following YF 17 DD (panels A and B), YF 17D SIV Gag _45-269_ (panels C and D) and YF 17D Δ IRES (panels E and F) virus immunization in C57BL/6 and BALB/c mice. The analysis was performed by Pearson’s correlation method.

In order to explore this hypothesis, we performed an ELISA protocol to measure IgG1 and IgG2a antibodies using the same serum samples from the neutralization test. Despite of being the method of choice to measure neutralizing antibodies, the PRNT_50_ does not necessarily correspond to the capacity of the antibody for neutralization *in vivo*, once it does not take into account the Fc participation in this process. Strikingly, as we can see in Figure 8 panels C, F and I, the IgG2a/IgG1 (T_H_1/T_H_2) ratio is not different between these two mice strains. In all groups immunized, the medians stayed around 1, showing a perfect equilibrium between T_H_1/ T_H_2 response, which is also a feature of immunization with YF viruses [4,34]. Nevertheless, the levels of neutralizing antibodies were positively correlated with IgG2a O.D. values in C57BL/6 mice (Figure 9, panels A, C and E) as well as in BALB/c mice (Figure 9 panels B, D and F) while there was no correlation with IgG1 O.Ds (data not shown), showing that IFN-γ might have a role during class switch for antibody production after YF 17D virus infection in mice. 

## Discussion

 In recent years, it has been suggested that early events after yellow fever vaccination are crucial to the development of adequate acquired immunity [2,5,6]. In our previous study we have described an early IFN-γ production after YF 17D and YF 17D recombinant virus vaccination in rhesus monkeys. This early IFN- γ production was attributed to γδ T cells and was shown to be a feature of yellow fever vaccination [8]. Corroborating this previous study, herein we have demonstrated that early IFN-γ production after yellow fever vaccination is a characteristic of murine infection and is much more pronounced in the C57BL/6 strain when compared to the BALB/c strain. These differences between the lineages were expected, since it has been previously demonstrated for other infections [12,13,35,36]. During the present study, γδ T cells producing IFN-γ were detected in draining lymph nodes as early as one day after YF 17D injection, mainly in C57BL/6 mice. Another study demonstrated that one day after injection of 17D virus in mice, it was possible to recover infected and activated dendritic cells (DCs) from draining lymph nodes [4]. Thus, we hypothesize that early IFN-γ response is triggered by virus replication in DCs. The infection by YF 17D attenuated virus activates murine DCs via multiple TLRs, including TLR-2, 7, 8 and 9 [4]. It is noteworthy that three of four TLRs involved in recognizing YF 17D virus have as major ligands nucleic acids, pointing to the dependence of replication for YF 17D virus recognition by DCs. In response to the TLRs engagement by virus products, DCs release several proinflammatory cytokines and chemokines, among then, IL-12 and IFN-α [4,37]. It is well known that genes associated with type I IFNs are early molecular signatures of successful YF 17D vaccination, with several interferon-stimulated genes (ISGs), such as interferon related factor 7 (IRF-7), being correlated with the onset of protective immunity [6]. Despite the demonstration that YF 17D is sensitive to some of these ISGs, the cells already infected by this virus are not sensitive to type I IFN, since the NS4B protein prevents STAT 1 phosphorylation, blocking type I IFN signaling inside the infected cell [38,39]. Although not being capable of responding to type I IFN, such cells retain the capacity to release it, signaling to other cells. In this way, it is possible that these proinflammatory mediators released by infected DCs, associated with products of virus replication, such as double stranded RNA, activate γδ T cells as well as NK cells to produce IFN-γ [40–44]. Conversely, human DCs co-cultured with YF 17D induced strong activation of Vγ9Vδ2 human T cells which release large amounts of IFN-γ, indicating a possible crosstalk between infected DCs and γδ T cells as well as NK cells [45]. 

In accordance with our hypothesis, for recombinant YF 17D viruses, we could also detect γδ^+^IFN-γ^+^ cells inside the lymph nodes but only three days after injection of C57BL/6 mice, pointing to the possibility that these viruses have a delay in replication in relation to the 17DD vaccine virus. It is possible that, with ongoing viremia, these γδ IFN-γ^+^ cells appear in the spleen only after three days of YF 17D inoculation. For recombinant viruses, despite the detection of these cells also by day 3, the magnitude of γδ^+^IFN-γ^+^ is similar to YF 17DD only by day 5. Besides γδ T cells, it was also possible to detect NK cells producing IFN-γ only in lymph nodes, but not in the spleen. This is supported by the observation by Wang and cols that in West Nile virus infection, γδ T cells were the main source of IFN-γ early after infection. In addition, they could not detect NK cells producing IFN-γ in the spleen [46].

IFN-γ is one of the most important molecules in the immune system with many different functions [28]. Early IFN-γ response has been shown as being of fundamental importance against several non-viral infectious agents (e.g. *Plasmodium falciparum*, *Listeria monocitogenes* and *Aspergillus* *sp*) as well as infection by other flaviviruses such as West Nile Virus. In addition, early IFN-γ production has already been considered as an important adjuvant property of the viral vector Canarypox ALVAC [46–50]. Once we have demonstrated that early IFN-γ is a strong feature of the mammalian immune response to YF virus, the question that rises is its relevance to the control of viral infection and quality of acquired response.

On one hand, the early IFN-γ response may be related to virus elimination from the organism. This hypothesis is supported by a previous study using a model of encephalitis by a neurotropic 17D strain. In this study they have shown that IFN-γ knockout mice previously immunized with the same strain was still able to survive the intracerebral challenge, but the virus loads were significantly higher when compared to wild type mice [51]. In this regard, recent works suggested that it is possible that IFN-γ exerts its antiviral activity by amplifying the signaling cascade triggered by type I IFNs. The authors have reported that IFN-γ, despite of being a less efficient activator of most ISGs than IFN-α, is a potent activator of Interferon Responsive Factor I (IRF-1), which leads to the production of more type I IFN, in a positive feedback cascade [52]. Thus, besides exercising direct antiviral effects, IFN-α/β acting synergistically with early IFN-γ would lead to the activation of other innate cells capable of assisting viral clearance.

It is well known that IFN-γ is one of the main activators of NK cells, γδ T cells and macrophages. Activated NK cells and γδ T cells are able to eliminate virus-infected cells through cytotoxic effects [53–55]. In fact, our previous work in humans showed that NK cells expressed augmented cytotoxic markers after YF 17DD vaccination. Furthermore, it has been reported that NK cells exhibit cytotoxicity against YF virus-infected cells in target organs (such as liver) through direct cytolysis as well as ADCC [7,56,57]. With regard to macrophages, IFN-γ expands the killing capacity of these cells by increasing phagocytosis and expression of key enzymes, such as inducible nitric oxide synthase (iNOS) [25]. Nitric oxide release by activated macrophages may be particularly important to YF virus elimination, since several flaviviruses are resistant to type I IFN anti-viral effects inside the infected cell. Accordingly, Dengue virus has been shown to be sensitive to NO action [58,59].. Noteworthy, the presence of activated monocytes has already been reported after human vaccination with YF 17DD[60].

On the other hand, increasing evidence suggests that the crosstalk between γδ T cells, NK cells and DCs inside the lymph nodes contribute to DC maturation and consequently to a better prime of specific T cells, leading to T_H_1 profile differentiation [22,23,45,61–63]. Hence, we have hypothesized that this early IFN-γ release by innate cells might directly influence the quality and magnitude of acquired response against the virus. In fact, CD4^+^ IFN-γ^+^ T cells, therefore T_H_1 cells, could be detected in lymph nodes as well as spleens of C57BL/6 mice immediately after the appearance of IFN-γ^+^ innate cells. We could also detect CD4^+^IFN-γ^+^ T cells in BALB/c mice, but with lower magnitude as in C57BL/6, particularly in lymph nodes, where C57BL/6 had a sustained IFN-γ^+^ CD4^+^ T cell response until day 7 post inoculation while the levels of these cells dropped in BALB/c mice. A similar profile was seen after T cell stimulation with 17D antigen, with higher percentages of IFN-γ- producing cells indicating a preference for the development of cells with effector profile in C57BL/6 mice [64]. This feature is of particular interest, since a study with knockout mice previously immunized and intracerebraly injected with a neurotropic strain of 17D virus has shown that specific CD4^+^ T cells are required to protect mice against lethal encephalitis caused by this virus [51]. Another recent study performed in humans vaccinated with 17D virus, described a wave of CD4^+^ IFN-γ^+^ antigen-specific cells two days after immunization, supporting our idea that innate cells may be involved in the priming of specific T_H_1 cells [65]. 

With regard to the response against the Gag insert, the CD4^+^ T cell specific response for 17D/ SIV Gag Δ IRES, which is a more stable virus [16] was better in C57BL/6 mice, including higher percentages of IL-2+ cells. These data suggest that CD4^+^T cells were better primed in C57BL/6 mice, once proinflammatory cytokines (mainly IFN-γ) in microenvironment and prolonged antigen exposure during priming (in this case, provided by a more stable recombinant virus) has been shown to increase the proportion of memory CD4^+^ T cells generated [66]. 

Moreover, the CD8^+^ T cells response in C57BL/6 mice is clearly of higher magnitude for all three viruses, showing a strong IFN-γ response after 17D stimulation. Regarding the response against the insert in recombinant viruses, once again the C57BL/6 model was more informative about the immunogenicity of both YF 17D SIV Gag_45-269_ and YF 17D SIV Gag Δ IRES viruses. The same differences in the quality and magnitude of CD8^+^ T cells response among C57BL/6 and BALB/c strains were described after dengue infection in mice and a strong and sustained IFN-γ response is correlated to a good prognosis after vaccination against Dengue virus [67]. 

Finally, we have investigated the differences in antibody production in these two mice lineages, since it has already been shown that the quality of B cell response can be programmed by innate cells, with a better quality of response being observed after privileging T_H_1 differentiation [29]. In fact, we observed higher titers of neutralizing antibodies and anti- YF total IgG in C57BL/6 mice after 17D immunization. For recombinant YF 17D viruses, it was not possible to see differences in neutralizing antibody production between the two lineages, maybe due to their over attenuation as compared to parental 17D virus [16,68,69]. The loss in fitness could lead to lower viral spread inside the lymph nodes and less virus coming to B cell zone. The quantity of whole virus inside B cell zone may be important to antigen-naïve B cell engagement and therefore, to neutralizing antibody production [70]. In this regard, we could show differences in anti YF total IgG detection, which production is not dependent on whole virus engagement, with the stable virus YF 17D/ SIV Gag Δ IRES having a trend towards a better performance than YF 17D/ SIV Gag _45-269_ (Figure 4). 

Another important aspect of antibodies against flaviviruses is the *in vivo* capacity of neutralization, which is directly linked to the IgG isotype [71]. Since IFN- γ may drive humoral immune responses, redirecting B cells from proliferation towards differentiation, promoting isotype switching to certain IgG subclasses [25], we have further explored the IgG isotypes produced after immunization with 17D and its recombinant derivatives. Surprisingly, it was not possible to see a higher IgG2a/ IgG1 in C57BL/6 mice when compared to BALB/c mice, what would represent a bias to T_H_1 response in this strain. Nevertheless, we did demonstrate that IgG2a (well known to be elicited by IFN-γ in mice) levels correlated to neutralizing antibody levels, while IgG1 did not. In this regard, only antibodies directed against the yellow fever Envelope (E) and Non-structural 1 (NS1) proteins belonging to IgG2a subclass, but not IgG1, protected mice against encephalitis after intracerebral challenge with YF-17D [72,73]. This fact is probably due to a higher capacity of IgG2a in activating the complement cascade [71].

 Altogether, the results presented here demonstrated that early production of IFN-γ is a feature of vaccination with YF 17D and recombinant YF 17D viruses, which occurs in mice, monkeys and humans. Moreover, we have shown, for the first time, that the magnitude of early IFN-γ production is related to a better T cell and antibody response and consequently, might influence the immunogenicity of YF 17D virus.

## Supporting Information

Figure S1 **The gating strategy employed to define the kinetics of IFN-γ production by γδ T cells, NK cells (DX-5+) CD4+ and CD8+ T cell populations *ex vivo* in the lymphnodes of immunized mice.** On the basis of forward scatter height (FSC-H) and area (FSC-a) properties we first excluded the doublets and bigger cell aggregates from the analysis. Then within the singlet gate we selected the lymphocyte population on the basis of the side (SSC-A) and forward scatter (FSC-A) properties of the cells. Within the lymphocyte gate we defined γδ T cells, NK cells (DX-5+) CD4+ and CD8+ T cell populations. Inside each cell populations, we gated IFN-γ+ cells. The representative figures are of draining lymphnodes from one C57BL/6 and one BALB/c animal immunized with YF 17DD virus after 5 days (γδ T cells, NK cells) and 7 days (CD4+ and CD8+ T cell) of inoculation.
(TIF)

Figure S2 **The gating strategy employed to define the kinetics of IFN-γ production by γδ T cells, NK cells (DX-5+) CD4+ and CD8+ T cell populations *ex vivo* in the spleens of immunized mice.** On the basis of forward scatter height (FSC-H) and area (FSC-a) properties we first excluded the doublets and bigger cell aggregates from the analysis. Then within the singlet gate we selected the lymphocyte population on the basis of the side (SSC-A) and forward scatter (FSC-A) properties of the cells. Within the lymphocyte gate we defined γδ T cells, NK cells (DX-5+) CD4+ and CD8+ T cell populations. Inside each cell populations, we gated IFN-γ+ cells. The representative figures are of spleens from one C57BL/6 and one BALB/c animal immunized with YF 17DD virus after 7 days of inoculation.
(TIF)

Figure S3 **The gating strategy employed to define YF and Gag specific CD4+ and CD8+ cell populations in the spleens of immunized mice.** The singlets gate, lymphocytes gate and CD4+ and CD8+ cells gates were designed as explained in S2 figure. Within the CD4+ (upper panel) gate or CD8+ (lower panel) we defined IFN-γ+ cells, IL-2+ cells and IFN-γ+IL-2+ cells . The representative figures are of spleens from one C57BL/6 and one BALB/c animal immunized with each virus.
(TIF)
