# Supplementary figures and images for: Early IFN-Gamma Production after YF 17D Vaccine Virus Immunization in Mice and Its Association with Adaptive Immune Responses
Source: PLoS One. 2013 Dec 6;8(12):e81953. doi: 10.1371/journal.pone.0081953 (PMC3855709; doi:10.1371/journal.pone.0081953)

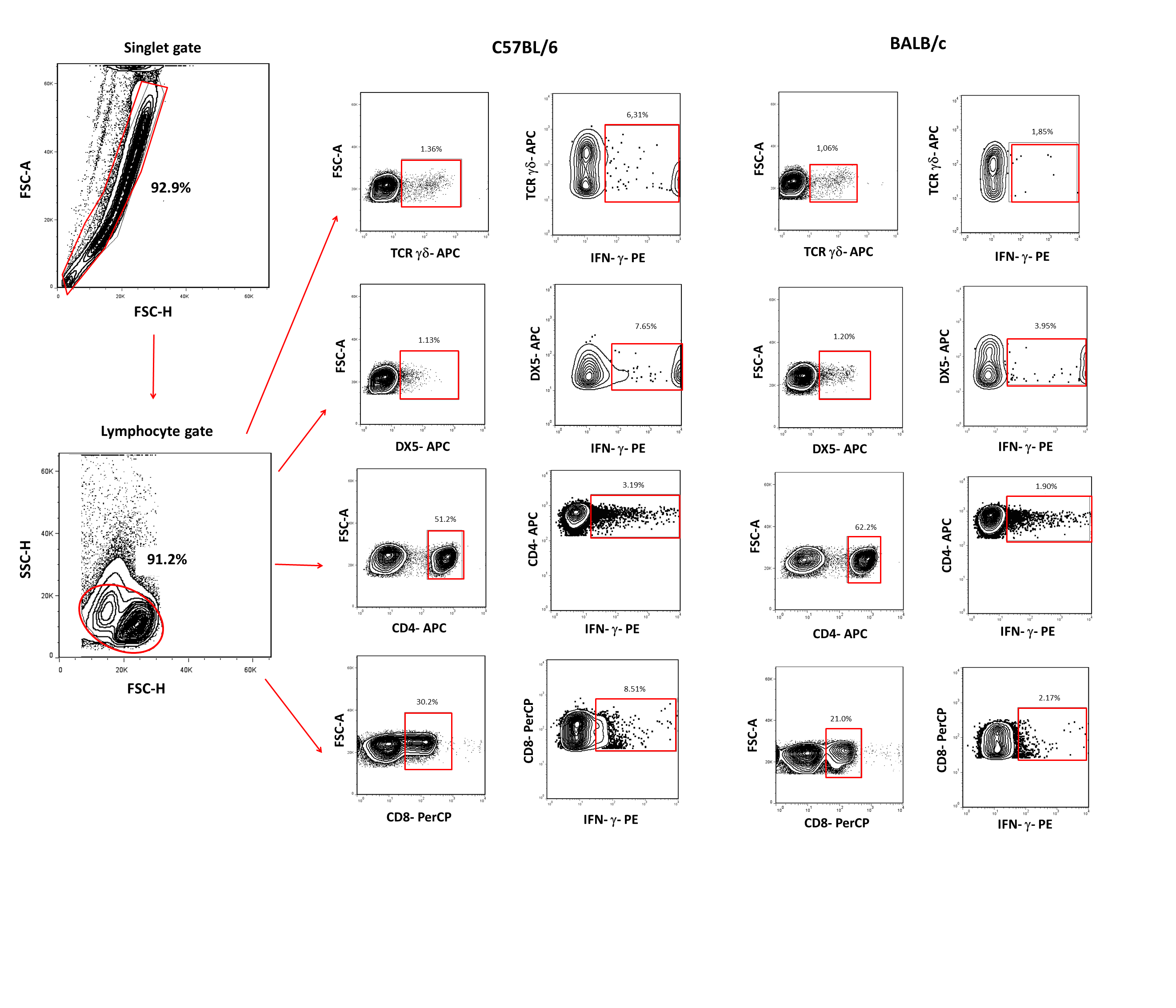

Supplement: Figure S1 — The gating strategy employed to define the kinetics of IFN-γ production by γδ T cells, NK cells (DX-5+) CD4+ and CD8+ T cell populations ex vivo in the lymphnodes of immunized mice. On the basis of forward scatter height (FSC-H) and area (FSC-a) properties we first excluded the doublets and bigger cell aggregates from the analysis. Then within the singlet gate we selected the lymphocyte population on the basis of the side (SSC-A) and forward scatter (FSC-A) properties of the cells. Within the lymphocyte gate we defined γδ T cells, NK cells (DX-5+) CD4+ and CD8+ T cell populations. Inside each cell populations, we gated IFN-γ+ cells. The representative figures are of draining lymphnodes from one C57BL/6 and one BALB/c animal immunized with YF 17DD virus after 5 days (γδ T cells, NK cells) and 7 days (CD4+ and CD8+ T cell) of inoculation. (TIF) [file pone.0081953.s001.tif]

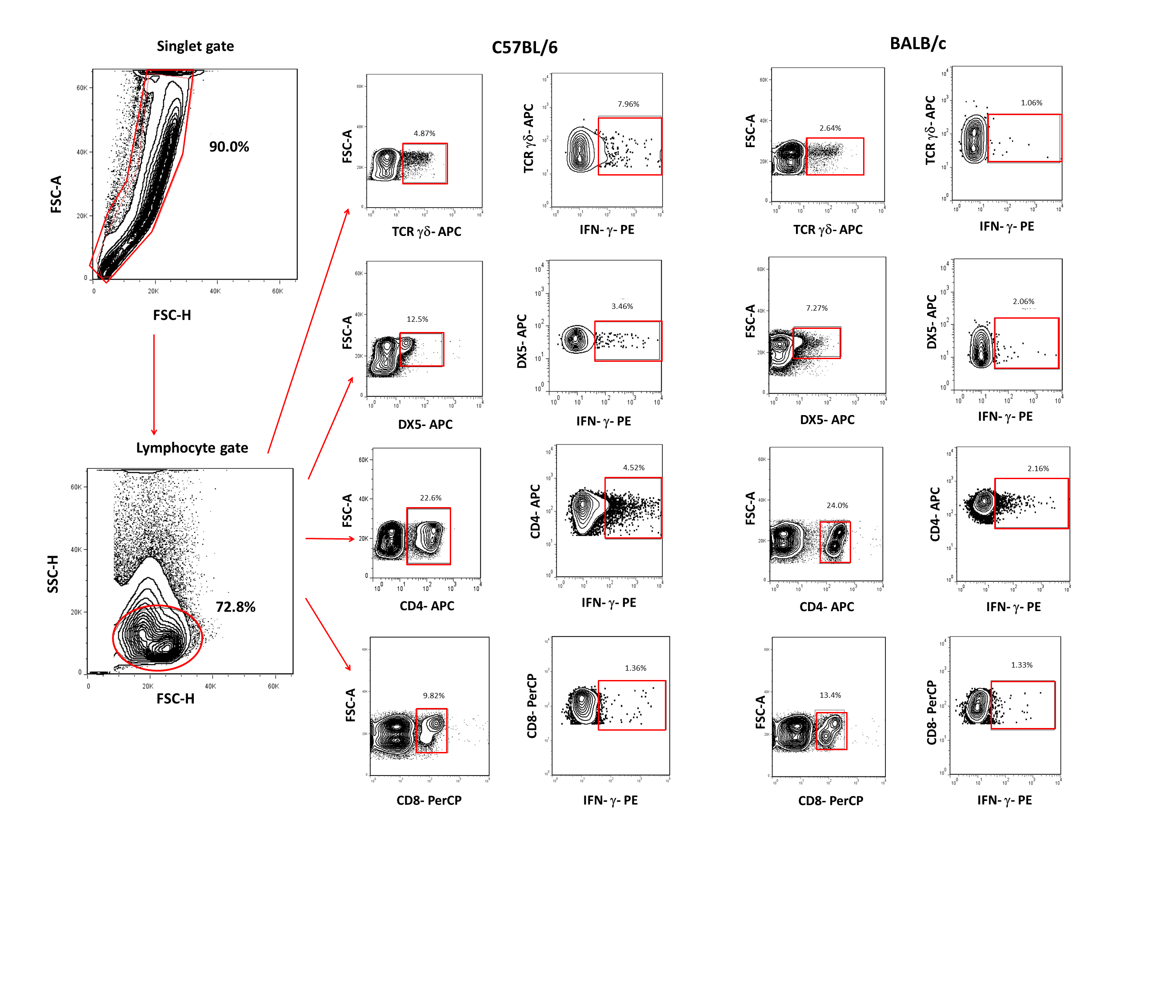

Supplement: Figure S2 — The gating strategy employed to define the kinetics of IFN-γ production by γδ T cells, NK cells (DX-5+) CD4+ and CD8+ T cell populations ex vivo in the spleens of immunized mice. On the basis of forward scatter height (FSC-H) and area (FSC-a) properties we first excluded the doublets and bigger cell aggregates from the analysis. Then within the singlet gate we selected the lymphocyte population on the basis of the side (SSC-A) and forward scatter (FSC-A) properties of the cells. Within the lymphocyte gate we defined γδ T cells, NK cells (DX-5+) CD4+ and CD8+ T cell populations. Inside each cell populations, we gated IFN-γ+ cells. The representative figures are of spleens from one C57BL/6 and one BALB/c animal immunized with YF 17DD virus after 7 days of inoculation. (TIF) [file pone.0081953.s002.tif]

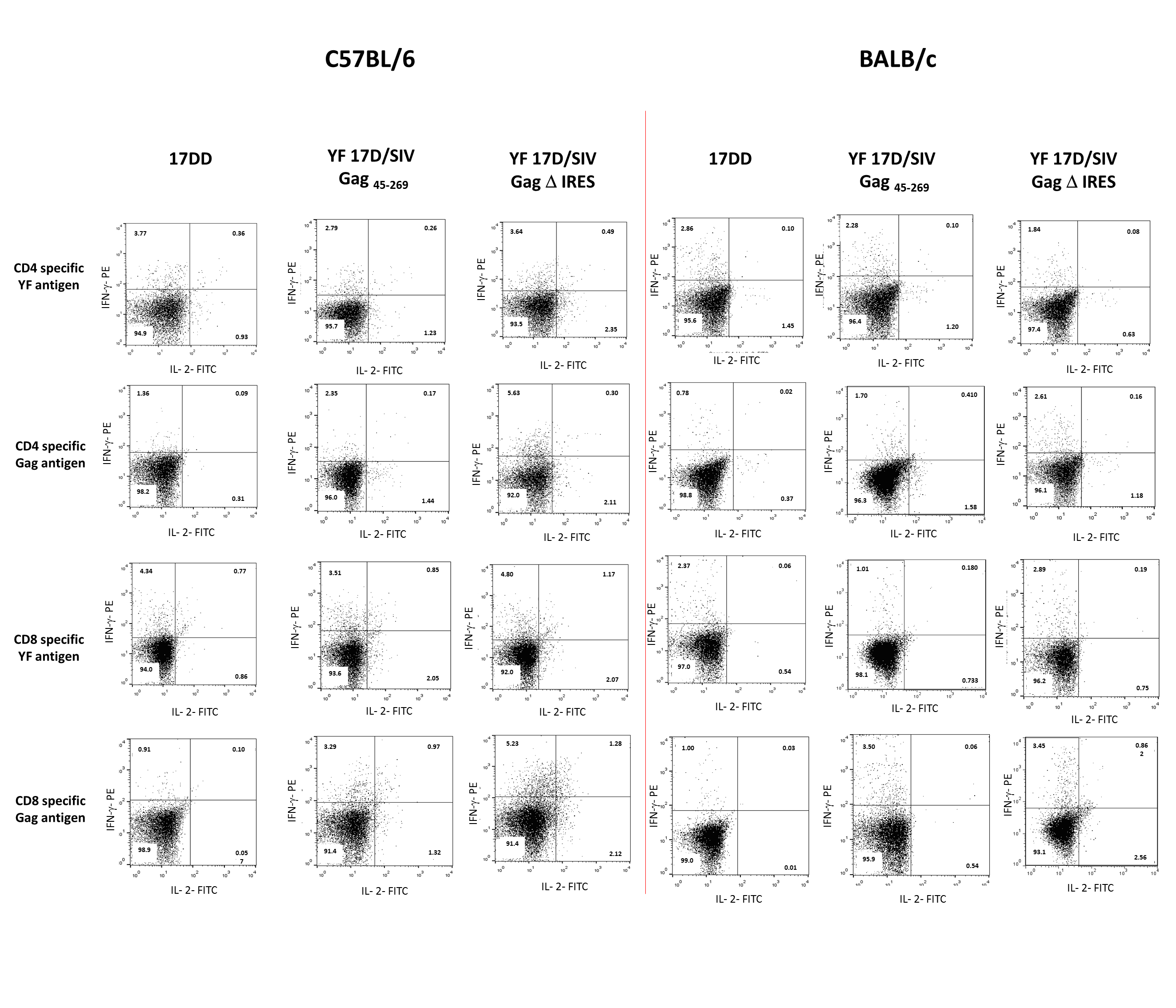

Supplement: Figure S3 — The gating strategy employed to define YF and Gag specific CD4+ and CD8+ cell populations in the spleens of immunized mice. The singlets gate, lymphocytes gate and CD4+ and CD8+ cells gates were designed as explained in S2 figure. Within the CD4+ (upper panel) gate or CD8+ (lower panel) we defined IFN-γ+ cells, IL-2+ cells and IFN-γ+IL-2+ cells . The representative figures are of spleens from one C57BL/6 and one BALB/c animal immunized with each virus. (TIF) [file pone.0081953.s003.tif]
